# Eye symptoms in patients with benign thyroid diseases

**DOI:** 10.1038/s41598-021-98232-0

**Published:** 2021-09-21

**Authors:** Nadia Sawicka-Gutaj, Paulina Ziółkowska, Klaudia Wojciechowska, Sara Shawkat, Agata Czarnywojtek, Wojciech Warchoł, Jerzy Sowiński, Ewelina Szczepanek-Parulska, Marek Ruchała

**Affiliations:** 1 Department of Endocrinology, Metabolism and Internal Medicine, Poznan University of Medical Sciences, 49 Przybyszewskiego St., 60-355 Poznan, Poland; 2 Department of Pharmacology, Poznan University of Medical Sciences, Poznan, Poland; 3 Department of Ophthalmology and Optometry, Poznan University of Medical Sciences, Poznan, Poland

**Keywords:** Endocrinology, Signs and symptoms

## Abstract

Thyroid diseases may cause a variety of functional and structural body changes, including eye and vision abnormalities, which can have a negative impact on a patient’s well-being. However, only a few studies on the impact of other benign thyroid diseases on the visual process are available in the literature. In this study, using the Polish version of the thyroid-specific quality of life (ThyPROpl) questionnaire, we aimed to determine the self-reported influence of benign thyroid diseases (e.g., nodular goiter, toxic nodular goiter, Graves’ disease, thyroid orbitopathy, Hashimoto’s thyroiditis, and surgical hypothyroidism) on patients’ eyes and vision. This was a prospective study. In total, 374 randomly selected euthyroid patients and 255 control subjects responded to the ThyPROpl questionnaire and the results were evaluated. Nearly 69% of the respondents reported that the most frequent condition was “reduced sight.” Men most often reported wet/tearing eyes (66%). The occurrence of eyelid sacks or swollen eyelids (64%), ophthalmalgia (62%), and eye dryness (61%) was marked almost as often. In total, 29% of the patients reported diplopia, and it was found to be most prevalent among those with thyroid orbitopathy. Other complaints were similarly prevalent among all the subgroups. A positive correlation was also observed between the scores of the “eye symptoms” and other ailments. Except for swelling around the lower eyelids, patients with thyroid diseases more frequently experienced all of the ocular complaints analyzed in this study compared with controls. This study showed that eye complaints are common in patients with benign thyroid diseases and ocular disturbances have a negative impact on the overall quality of life of patients.

## Introduction

Thyroid diseases are common endocrine disorders and may cause a variety of functional and structural body changes, including eye and vision abnormalities, which have a negative impact on a patient’s well-being^1,2^.

Thyroid-associated ophthalmopathy (TAO)—also known as thyroid eye disease, Graves’ orbitopathy, or Graves’ ophthalmopathy (GO)—is an inflammatory disease of the eye and orbital tissues^2^ and is reported to be one of the most common causes of ocular myopathy in adults. Generally, TAO is associated with Graves’ disease; however, regardless of the thyroid function, it may also occur in patients with chronic lymphocytic thyroiditis (Hashimoto’s disease)^3,4^. The clinical signs and symptoms characteristic of TAO are double vision, proptosis, lid retraction, restrictive extraocular myopathy, optic neuropathy, and inflammatory ocular surface disorders. However, as the pathogenesis of TAO is not fully understood, the therapeutic options are limited^5–8^.

TAO is rarely reported and is less known^3,9^ in the case of chronic lymphocytic thyroiditis or Hashimoto’s disease. Moreover, patients with Hashimoto’s disease may develop eye changes similar to those observed in patients with TAO, such as symptoms related to dry eye syndrome. These symptoms cause serious discomforts such as dryness, burning, grittiness, and eye irritation that increase during the daytime^3^. If dry eye syndrome-related symptoms are left untreated, then they may damage the eyes, thereby resulting in vision impairment or rarely in the loss of vision^10,11^. These changes when occur in long term lead to mood or sleep disorders^1,12^. It should also be emphasized that some clinical conditions may mimic TAO and, therefore, each patient with a suspicion of TAO should be differentially diagnosed to rule out non-thyroid etiology^13,14^.

Nearly 80% of all sensory processing that occurs in the body is directly related to the information that is transmitted from the eyes to the brain. Therefore, any impairment in vision can lead to functional disability and greatly affect an individual’s personal and social behavior^15,16^. Numerous studies have confirmed that TAO can cause a significant decrease in the quality of life (QoL), and thyroid dysfunction leads to further worsening of a patient’s well-being. However, only a few studies on the impact of other benign thyroid diseases on the visual process are available in the literature. Further, the objective methods used for an eye examination often differ based on an individual’s perception and self-esteem—the factors that affect the daily life of an individual the most. Therefore, in this study, using the Polish version of the thyroid-specific QoL (ThyPROpl) questionnaire, we aimed to determine the self-reported influence of benign thyroid diseases on the eyes and vision of patients^17^. We analyzed the eye symptoms reported by patients in euthyroid state and assessed the potential relationship between the reported complaints and the overall QoL—clinical as well as biochemical parameters. We also compared the responses of the patients with those of the control group (adults with no history of thyroid disease).

## Patients and methods

This was a prospective study. In total, 374 euthyroid patients with benign thyroid diseases were randomly selected from the Department of Endocrinology, Metabolism and Internal Diseases, and outpatient clinic of the Clinical Hospital of the Poznan University of Medical Sciences. They were asked to complete the ThyPROpl questionnaire, and the results were examined. Each patient participating in the study was assigned to one of the following six subgroups in order to examine the subjective feelings and differences between patients struggling with various thyroid problems: 1, nodular goiter; 2, toxic nodular goiter; 3, Graves’ disease; 4, thyroid orbitopathy; 5, chronic lymphocytic thyroiditis; and 6, surgical hypothyroidism. Exclusion criteria were the presence of other chronic diseases, including type 2 diabetes, hypertension, other autoimmune diseases, or cancers potentially affecting vision and QoL. Laboratory tests (TSH, thyroid-stimulating hormone; fT3, free triiodothyronine; fT4, free thyroxine; TRAb, TSH-receptor antibodies; TPOAb, anti-thyroperoxidase antibodies; TgAb, thyroglobulin antibodies) were performed on the same day. Each patient underwent a physical examination. Patients with thyroid orbitopathy underwent an ophthalmological consultation and had a magnetic resonance imaging of orbits. Patients with hypothyroidism were treated with levothyroxine, while those with hyperthyroidism (e.g., Graves’ disease and toxic nodular goiter) were treated with thiamazole. The control group consisted of 255 healthy people and answered questions from the nine scales of ThyPRO questionnaire without specific attribution to thyroid disease. In addition, sociodemographic data were collected from all the respondents and TSH levels were reported by healthy participants.

ThyPRO is a validated thyroid-specific QoL questionnaire^18,19^. We used its Polish version (ThyPROpl), which was developed according to the standard methodology used for the translation of patient-reported outcomes^17^. It contains 85 questions summarized in 13 scales, measuring various aspects of QoL. Patients are asked to mark their answers on the five-point Likert scale: 0, not at all; 1, a bit; 2, on average; 3, quite strongly; and 4, very much. The reference period is 4 weeks. If more than half of the questions on the scale are answered, then the number of points is divided by the total maximum number for the subscale as a whole and the obtained result is linearly transformed into a scale of 0–100.

The domain assessing eye symptoms and vision disorders includes the following eight questions about visual problems that are often associated with thyroid diseases:

- Have you had wet or tearing eyes?
- Have you had bags under your eyes or swollen eyelids?
- Did you have a feeling of dryness or a feeling of sand under the eyelids?
- Have you had eye problems?
- Have you felt pressure within (or behind) the eyeballs?
- Have you had double vision?
- Did your eyes ache?
- Have you felt hypersensitivity to light?

### Ethics

The study was approved by the Ethical Committee of Poznan University of Medical Sciences, and all patients signed informed consent to participate in the study. All the methods used in the study were performed in accordance with the relevant guidelines and regulations.

### Statistical analysis

Statistical analysis was performed using the Statistica program (universal system for statistical data analysis, version 13.1). The results between men and women were compared using the Mann–Whitney test. The analyzed parameters were compared between all groups using the ANOVA/Kruskal–Wallis test, and the responses of study subgroups and healthy controls were compared using Fisher’s exact test. The Shapiro–Wilk test was applied to investigate the normality of data distribution. Therefore, the data did not follow a normal distribution, and were presented as medians and as lower and upper quartiles (Tables 1, 2). Potential correlations between the parameters were assessed by Spearman’s *R* test. A *p*-value less than or equal to 0.05 was considered statistically significant.

**Table 1:** Clinical data and laboratory test results of patients participating in the study.

	Number of available data (N)	Percentage of available data (%)	Median	IQR
Men/women	70/304	19/81	–	–
Age, years	292	78	50.0	39–64
TSH, mIU/L	280	75	0.6	0.1–1.6
fT3, pmol/L	151	40	5.0	4.1–5.0
fT4, pmol/L	206	55	17.0	14.3–20.1
TRAb, IU/L	56	15	1.2	0.3–4.3
TPOAb, IU/ml	100	27	22.0	8.0–105.5
TgAb, IU/ml	82	22	35.5	10.0–64.0

*fT3* free triiodothyronine, *fT4* free thyroxine, *IQR* interquartile range, *N* the number of valid answers, *TgAb* thyroglobulin antibodies, *TPOAb* anti-thyroperoxidase antibodies, *TRAb* TSH-receptor antibodies, *TSH* thyroid-stimulating hormone.

**Table 2:** Response of patients with benign thyroid diseases to the “eye symptoms” section of the ThyPROpl questionnaire.

Diagnosis	N (%)	Median	IQR
Nodular goiter	81 (22)	16	9–38
Toxic nodular goiter	74 (20)	21	9–34
Graves’ disease	60 (16)	25	9–44
Thyroid orbitopathy	12 (3)	66	22–78
Chronic lymphocytic thyroiditis	67 (18)	19	9–38
Surgical hypothyroidism	78 (21)	19	9–38

*IQR* interquartile range, *N* the number of valid answers.

## Results

### Study subjects

The study involved 374 patients, 81% of whom were women. Clinical data are provided in Table 1.

**Figure 1:**
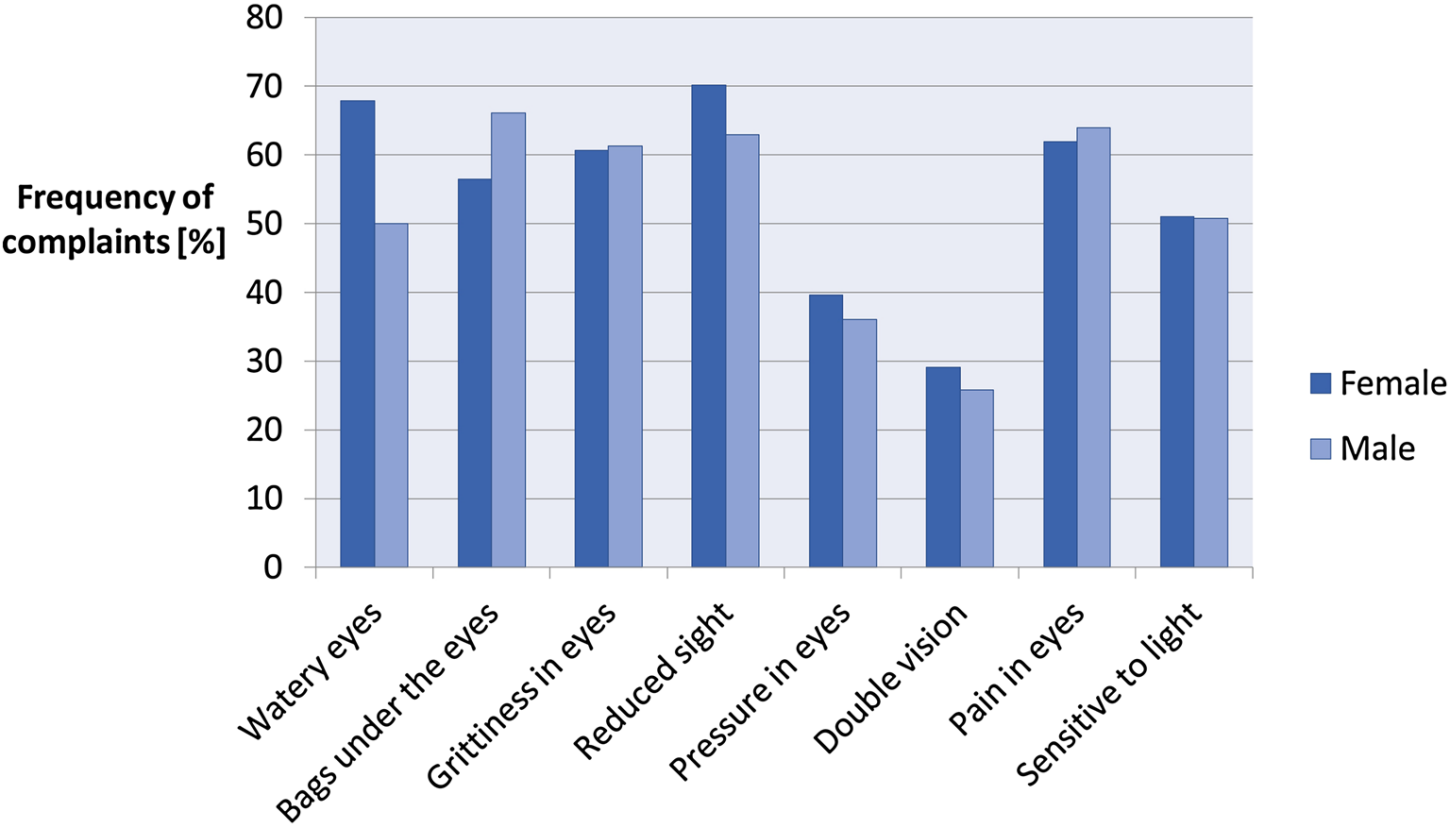
The prevalence of eye symptoms in patients with thyroid diseases based on the results of ThyPROpl questionnaire among women and men.

Figure 1 shows the prevalence of individual ocular complaints in male and female patients. The most frequently reported condition by the patients was “reduced sight”—as many as 69% of respondents received a score equal to or greater than 1 point on the Likert scale. Nearly 66% of the men reported wet/tearing eyes most often. The patients also frequently reported the occurrence of eyelid sacks or swollen eyelids (64%), ophthalmalgia (62%), and eye dryness (61%). However, they less likely experienced double vision, but still, nearly one-third of them reported diplopia (29%). For individual questions, the differences in results did not exceed 10 percentage points for both men and women.

Patients from each of the six study groups had similar TSH concentrations (*p* = 0.1368), but they showed differences in the levels of thyroid autoantibodies (TRAb: *p* = 0.0004, TPOAb: *p* = 0.003, TgAb: *p* = 0.0009). Patients with Graves’ disease and thyroid orbitopathy had the highest levels of TRAb, while patients with chronic lymphocytic thyroiditis and surgical hypothyroidism had higher levels of TPOAb.

Table 2 shows the scores of eye domain for each subgroup. Patients with thyroid orbitopathy showed the highest percentage of vision problems. In addition, patients with thyroid orbitopathy (subgroup 4), nodular goiter, and toxic nodular goiter responded differently to ocular symptoms, which was statistically significant, as well as patients with chronic lymphocytic thyroiditis and surgical hypothyroidism (subgroups 1, 2, 5, and 6). In the case of subgroup 3 (Graves’ disease), the difference was not statistically significant.

The responses of patients to specific questions in the section “ocular complaints” were also compared between subgroups. Patients with thyroid orbitopathy more frequently reported double vision (*p* = 0.0018), while other complaints were similarly prevalent among all subgroups.

The results showed a positive relationship between the scores of the “eye symptoms” scale and the results of all other domains of the ThyPROpl questionnaire (Table 3).

**Table 3:** Results of the correlation of scores of “eye symptoms” section with other scales of ThyPROpl questionnaire (sugerowane usunięcie – wg recenzenta nie pasuje do koncepcji artykułu).

A pair of variables	N	*R* Spearman	*p*-value
Eye symptoms and cognitive complaints	371	0.49	< 0.0000001
Eye symptoms and anxiety	370	0.45	< 0.0000001
Eye symptoms and depressivity	368	0.39	< 0.0000001
Eye symptoms and impaired social life	372	0.36	< 0.0000001
Eye symptoms and impaired daily life	369	0.42	< 0.0000001
Eye symptoms and cosmetic complaints	369	0.46	< 0.0000001
Eye symptoms and overall quality of life	364	0.40	< 0.0000001

*N* the number of valid answers, *R Spearman* Spearman’s *R* test result.

The results showed no correlations between the scores of the “eye symptoms” scale, antibodies (TRAb, TgAb, TPOAb), TSH, fT3, fT4, and the age of the subjects in all the groups (*p* > 0.05).In addition, further analyses among subgroups did not show any correlations between the parameters analyzed and eye complaints (*p* > 0.05). Eye symptoms, cognitive complaints, and daily life scores were found to be similar for both men and women.

### General population sample

A total of 255 healthy people (207 women and 48 men) returned a completed ThyPROpl questionnaire. The median age of the control group was 54 years (interquartile range: 44.3–60.7 years). The sex distribution and age of the control group resembled those of patients with thyroid disorders (*p* = 0.5639 and *p* = 0.3983, respectively). The mean TSH concentrations were 1.77 ± 0.75 µIU/ml. Nearly 51% of the healthy controls most frequently reported “bags under the eyes,” while only 7.8% reported double vision. The comparison of results obtained revealed that patients with thyroid diseases more frequently experienced all of the ocular complaints analyzed in this study, except swelling around the lower eyelids, which was more common in patients with Graves’ disease and chronic lymphocytic thyroiditis. The results obtained are given in Table 4.

**Table 4:** Comparison of prevalence of particular ocular complaints between patients with thyroid disorders and healthy controls.

Study group	Nodular goiter N (81)	Toxic nodular goiter N (74)	Graves’ disease N (60)	Thyroid orbitopathy N (12)	Chronic lymphocytic thyroiditis N (67)	Surgical hypothyroidism N (78)	Controls
Watery eyes, N (%) *p*	42 (52.5) *p* = 0.002	47 (63.5) *p* = 0.000006	40 (66.7) *p* = 0.000004	10 (90.9) *p* = 0.0002	35 (52.2) *p* = 0.007	39 (51.3) *p* = 0.007	85 (33.3)
Bags under the eyes, N (%) *p*	47 (60.3) *p* = 0.157	44 (59.5) *p* = 0.234	43 (71.7) *p* = 0.004	9 (81.8) *p* = 0.063	45 (67.2) *p* = 0.019	48 (62.3) *p* = 0.090	130 (51)
Grittiness in eye, N (%) *p*	47 (58.8) *p* = 0.007	47 (63.5) *p* = 0.0008	39 (66.1) *p* = 0.0005	9 (81.8) *p* = 0.010	37 (55.2) *p* = 0.038	43 (56.6) *p* = 0.018	104 (40.8)
Reduced sight, N (%) *p*	54 (67.5) *p* < 0.000001	49 (66.2) *p* < 0.000001	40 (66.7) *p* < 0.000001	8 (72.7) *p* = 0.005	45 (68.2) *p* < 0.000001	56 (73.7) *p* < 0.000001	76 (29.9)
Pressure in eyes, N (%) *p*	25 (30.9) *p* = 0.003	27 (37.5) *p* = 0.0001	26 (44.1) *p* = 0.000004	7 (70) *p* = 0.0002	29 (43.9) *p* = 0.000002	28 (36.8) *p* = 0.0001	39 (15.3)
Double vision, N (%) *p*	20 (25) *p* = 0.001	17 (23.3) *p* = 0.0006	23 (38.3) *p* < 0.000001	9 (81.8) *p* < 0.000001	20 (30.3) *p* = 0.000007	17 (22.1) *p* = 0.001	20 (7.8)
Pain in eyes, N (%) *p*	44 (55) *p* = 0.014	46 (63) *p* = 0.0003	41 (68.3) *p* = 0.00004	8 (72.7) *p* = 0.031	41 (62.1) *p* = 0.0008	46 (60.5) *p* = 0.001	99 (38.8)
Sensitive to light, N (%) *p*	37 (47.4) *p* = 0.0002	33 (45.8) *p* = 0.0007	36 (62.1) *p* < 0.000001	9 (81.8) *p* = 0.0002	34 (52.3) *p* = 0.00003	36 (47.4) *p* = 0.0003	63 (24.7)

*N* number of patients, *p*, *p*-value.

## Discussion

This study, using the ThyPROpl questionnaire, aimed to analyze the self-reported thyroid-related ocular complaints by patients with benign thyroid diseases and the impact of these diseases on patients’ QoL. Generally, eye changes are usually associated with Graves’ disease; therefore, previous studies that analyzed ocular symptoms in thyroid diseases mainly focused on patients with Graves’ disease and Graves’ orbitopathy^20^. The novelty of our study was that we investigated and compared ocular complaints reported by patients with various benign thyroid diseases in the euthyroid state. Therefore, the ThyPROpl questionnaire proved to be an effective tool for us. ThyPROpl questionnaire helped us to explore the patients’ perception and self-esteem, which particularly affected their QoL. Furthermore, we compared our results with a sex- and age-matched healthy control group.

Both generic and disease-specific questionnaires showed significant worsening of well-being in patients with Graves’ orbitopathy^21–23^. Furthermore, patients with thyroid dysfunctions, chronic lymphocytic thyroiditis, and nodular goiter showed a decrease in QoL^24–26^. However, in patients with benign thyroid disorders, surgical treatment or therapy aiming to restore thyroid function showed a beneficial impact on the QoL, but in some patients, it did not show any normalization in the patient-reported well-being even after several years of diagnosis^27–29^.

We observed that visual complaints are common in patients with benign thyroid diseases, which affect the various aspects of vision. Nearly 70% of patients with thyroid disease experienced eye problems, which may be interpreted as a decrease in visual acuity, but they also experienced other impairments such as cosmetic abnormalities. Of the eight complaints included in this study, as many as six were reported by more than half of the patients, and this shows that they should be referred to an ophthalmologist or optometrist for an accurate diagnosis. Almost equally often, patients reported swelling around their lower eyelids, grittiness, and pain in the eyes. Over 50% of patients also reported watery eyes (the most common ailment among men and patients with orbitopathy) and photosensitivity. These complaints may indicate dry eye syndrome, which is described as the most common cause of discomfort in TAO, and up to 85% of patients reported that they had dry eye syndrome. Moreover, the prevalence of ocular complaints was significantly higher in patients with thyroid diseases than in the general population.

It is difficult to determine whether thyroid-attributed ocular complaints experienced by patients are indeed caused by thyroid diseases. To some extent, autoimmune pathogenesis underlying Graves’ disease and chronic lymphocytic thyroiditis may explain the ocular and vision impairment reported by the patients; however, it is relatively difficult to explain the common eye changes experienced by patients with nodular goiter. Studies that have analyzed visual disturbances associated with thyroid nodular disease are scarce. However, in studies that have focused on the QoL of patients with Graves’ disease and toxic nodular goiter, patients with toxic nodular goiter, for unknown reasons, also experienced “eye Symptoms”^23^. The authors deliberated whether these results were directly related to thyroid disease or not. However, immunological markers that might be involved in the development of nodular goiter may explain the eye symptoms in those patients^30^.

Certainly, future research should focus on discovering specific mechanisms linking eyes symptoms and thyroid immunogenicity.

The prevalence of individual complaints was observed to be different in patient subgroups. As suspected, patients with Graves’ orbitopathy frequently complained of problems. Patients with Graves’ disease and chronic lymphocytic thyroiditis also complained frequently, which may suggest the role of ongoing autoimmune processes or unrecognized mild orbitopathy in these groups. Although thyrotropin receptors act as primary autoantigens in the pathogenesis of TAO in patients with Graves’ disease, TRAbs are not observed in patients with chronic lymphocytic thyroiditis^8^. However, in chronic lymphocytic thyroiditis, antibodies specific to ocular muscle antigens, namely calsequestrin, collagen XIII, flavoprotein, and protein G2s, may occur^31–33^.

Differences in the response of patients in subgroups to “ocular complaints” and differences in the results of laboratory tests were examined. The results revealed no differences in responses between the six subgroups with regard to all ailments except for double vision, which was mainly reported by patients with Graves’ orbitopathy. Double vision, which involves oculomotor muscles, was the least reported complaint; however, every third patient was bothered by double vision. This might suggest that in a significant proportion of patients with Graves’ disease, orbitopathy was misdiagnosed. Another explanation for the relatively high prevalence of self-reported double vision might be the fact that patients incorrectly used this term.

Further, in this study, the potential relationships between the reported complaints and thyroid metabolism were analyzed. Because euthyroidism was an inclusion criterion, no statistically significant differences in TSH were observed between the subgroups, but antibody tests showed significant differences. Also, no correlation between the results of laboratory tests, age of the subjects, and responses to the “ocular complaints” scale was observed. Moreover, a correlation was observed between the scale outcomes and TRAb levels. Previous studies have shown a negative correlation between TPOAb, TgAb, and QoL^34–36^. In addition, TSH concentration was identified to be an important factor affecting the QoL of GO patients^37,38^. The results of GO-QoL questionnaire showed that younger patients had significantly better cognitive functioning and daily life^25^. However, elderly patients had significantly fewer complaints with regard to appearance (cosmetics), which also contributes toward QoL impairment^39^. Furthermore, young Taiwanese adults showed lower scores on the scale with regard to appearance^40^.

This study showed no statistically significant differences between the responses of men and women to the questionnaires. However, to date, it has been demonstrated that gender has a significant effect on the well-being of a patient with thyroid diseases. Studies have been conducted in different populations, which may also be relevant. For example, Ponto et al. and Riguetto et al. showed better QoL in male patients, whereas Lin et al. showed better QoL in female patients^22,25,40^. The results obtained by these authors and the results obtained by us in this study with regard to the effects of gender on the well-being of patients with thyroid diseases indicate that there may be large discrepancies in this parameter in individual studies.

We have also proved that the patients’ overall QoL was negatively affected by ocular complaints. A correlation study showed a positive relationship between the scores of the “ocular complaints” scale and those of the remaining symptoms mentioned in the questionnaire, such as depression and anxiety, disorders in everyday and social life, cognitive disorders, cosmetic complaints, and general QoL. This means that subjective visual disturbances worsen the QoL of patients, and solving such visual problems could improve their well-being^2^.

It would be worthwhile if the survey is reconducted by applying appropriate ophthalmic and optometric tests and adequate sources (e.g., spherical or prismatic glasses and moisturizing drops) to improve vision. This would allow examining the impact of nonsurgical methods in improving the patients’ QoL with regard to comfort and vision function^41^.

## Conclusions

The results of this study demonstrate that eye complaints in patients with benign thyroid diseases are common and that they affect the overall QoL of the patients. So, a multidisciplinary approach that comprises an endocrinologist, an ophthalmologist, an optometrist, and a psychologist is necessary for patients with thyroid diseases to identify co-existing ocular problems and implement appropriate therapy.
